# Association of Life’s Essential 8 with Hepatic Fibrosis, MASLD, and MetALD in the Framingham Heart Study

**DOI:** 10.3390/nu18081276

**Published:** 2026-04-17

**Authors:** Alejandro Campos, Tianyu Liu, Brenton Prescott, Jiantao Ma, Madeleine G. Haff, Maura E. Walker, Arpan Mohanty, Vanessa Xanthakis

**Affiliations:** 1 Department of Medicine, Boston University Chobanian & Avedisian School of Medicine and Boston Medical Center, Boston, MA 02118, USA; 2 Department of Epidemiology, Boston University School of Public Health, Boston, MA 02118, USA; 3 Section of Preventive Medicine and Epidemiology, Department of Medicine, Boston University Chobanian & Avedisian School of Medicine, Boston University, Boston, MA 02218, USA; 4 Nutrition Epidemiology and Data Science, Friedman School of Nutrition Science and Policy, Tufts University, Boston, MA 02111, USA; 5 Section of Gastroenterology, Department of Medicine, Boston University Chobanian & Avedisian School of Medicine and Boston Medical Center, Boston, MA 02118, USA; 6 Department of Health Sciences, Sargent College of Health and Rehabilitation, Boston University, Boston, MA 02215, USA; 7 Boston University and NHLBI’s Framingham Study, Framingham, MA 01702, USA; 8 Department of Biostatistics, Boston University School of Public Health, Boston, MA 02118, USA

**Keywords:** metabolic dysfunction-associated steatotic liver disease, liver fibrosis, cardiovascular health

## Abstract

**Background:** Metabolic dysfunction-associated steatotic liver disease (MASLD), metabolic dysfunction and alcohol-associated liver disease (MetALD), and related fibrosis are increasingly prevalent conditions. The relation of the American Heart Association’s (AHA) cardiovascular health (CVH) metric Life’s Essential 8 (LE8) with MASLD, MetALD, and hepatic fibrosis remains unclear. We aimed to investigate the associations of CVH with MASLD, MetALD, and hepatic fibrosis. **Methods:** We defined significant hepatic fibrosis as a liver stiffness ≥8.2 kPa measured by vibration-controlled transient elastography. MASLD was defined as steatosis (controlled attenuation parameter of ≥274 dB/m) with ≥1 cardiometabolic risk factor and mild alcohol intake (≤140 g/week [women]; ≤210 g/week [men]). MetALD was defined as steatosis with ≥1 cardiometabolic risk factor and moderate alcohol intake (141–350 g/week [women]; 211–420 g/week [men]). Data from 2962 participants in the Framingham Heart Study (mean age 59 years, 57% women) were used in multivariable-adjusted logistic regression models, accounting for demographic and clinical covariates to relate CVH and liver outcomes. **Results:** Our study included 2704 participants with mild and 258 with moderate alcohol use. MASLD and MetALD prevalence was 34% and 40%, respectively, and 9% had significant hepatic fibrosis. Each 10-point increase in LE4 score (composite of diet, sleep health, physical activity, and smoking) was associated with 16% lower odds of MASLD (Odds Ratio [OR] 0.84; 95% CI: 0.80–0.90; *p* < 0.001) but not MetALD. Each 10-point increase in LE8 score was associated with 17% lower odds of hepatic fibrosis (OR 0.83; 95% CI: 0.78–0.89; *p* < 0.001). **Conclusions:** Better CVH is related to lower odds of MASLD and significant hepatic fibrosis.

## 1. Introduction

Metabolic dysfunction-associated steatotic liver disease (MASLD), formerly known as non-alcoholic fatty liver disease (NAFLD), is the most prevalent chronic liver disease globally and a major public health concern [1,2]. The recent redefinition of MASLD emphasizes the central role of metabolic dysfunction and insulin resistance in its pathogenesis; an MASLD diagnosis requires cardiometabolic risk factors (CMRFs) including elevated body mass index (BMI), waist circumference (WC), blood pressure (BP), triglycerides (TG), blood glucose, and low high-density lipoprotein (HDL) cholesterol in individuals with steatosis and mild alcohol intake (≤140 g/week for women and ≤210 g/week for men) [3]. In people with steatotic liver disease, hepatic fibrosis is the single most important predictor of adverse liver-related [4,5] and cardiovascular events [5,6] and all-cause mortality [5,7,8]. Because alcohol use contributes to steatosis, accelerates hepatic fibrosis progression [9], and frequently coexists with CMRFs, the term metabolic and alcohol-associated liver disease (MetALD) was introduced to describe individuals with steatosis, moderate alcohol intake (141–350 g/week for women and 211–420 g/week for men), and concomitant CMRFs [3].

To promote better cardiovascular health (CVH), the American Heart Association (AHA) initially introduced Life’s Simple 7 (LS7) score to assess and track CVH and later updated this framework to the Life’s Essential 8 (LE8) score, which provides a more comprehensive and actionable measure of CVH by adding sleep health information. LE8 includes four health behaviors, i.e., diet, physical activity, nicotine exposure, sleep health, and four health factors (BMI, blood glucose, blood pressure, and non-HDL cholesterol) [10,11,12]. The CMRFs that define MASLD and MetALD are also key components of the CVH scores, which are strongly related to the development of CVD and all-cause mortality. While prior studies have reported inverse associations of CVH with MASLD and hepatic fibrosis [13,14,15,16,17], few studies have used well-characterized CVH data, imaging-based noninvasive assessment of hepatic steatosis and fibrosis, and evaluated associations with MetALD. Further, there is limited evidence on potential modification of these associations by genetic risk [18,19,20,21,22].

To address these gaps, we used data from the Framingham Heart Study (FHS), a well-characterized, community-based group, and investigated: (a) the associations of a composite score including the four health behaviors (LE4) with MASLD and MetALD to avoid overlap with the CMRFs used for defining MASLD and MetALD; given that the health factor components of LE8 (BMI, blood glucose, blood pressure, and non-HDL cholesterol) are by definition embedded within the diagnostic criteria for these conditions, which would result in redundant covariate adjustment if the full LE8 score were used; and (b) the relation of LE8 with hepatic fibrosis. We also explored potential effect modifications of these relations by a genetic risk score (GRS) for hepatic steatosis and fibrosis. We hypothesized that more favorable LE4 and LE8 scores are associated with lower odds of MASLD, MetALD, and hepatic fibrosis, and favorable CVH attenuates the adverse effects of genetic risk on these outcomes.

## 2. Materials and Methods

### 2.1. Participants

**Figure 1:**
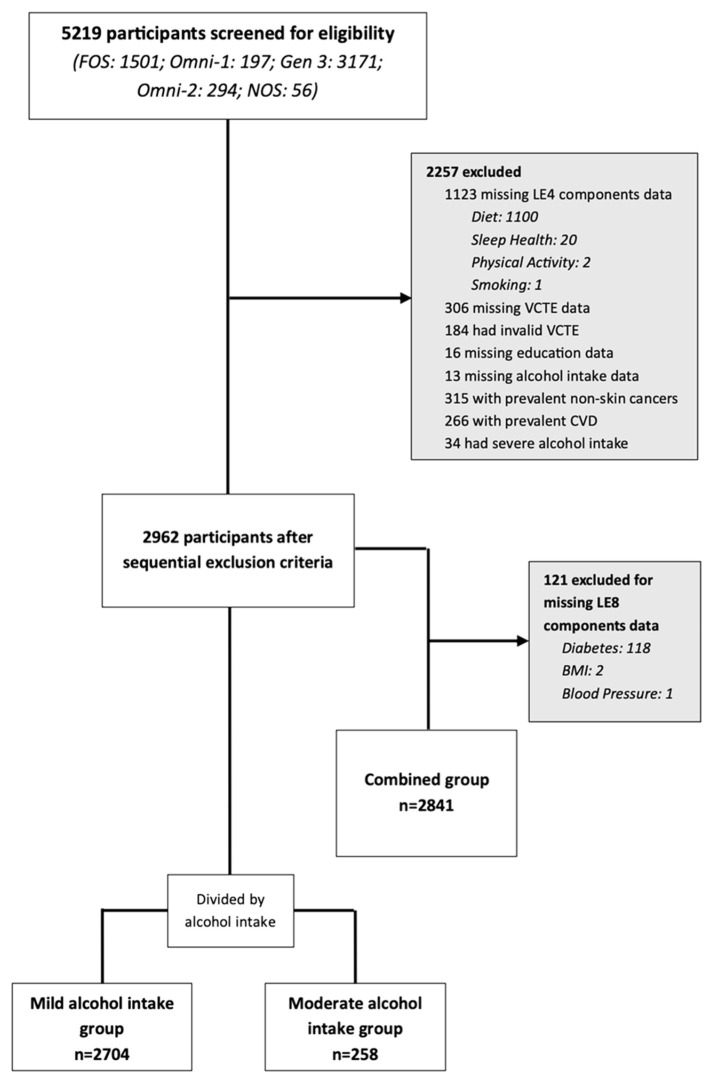
Study Groups Derivation. FOS: Framingham Offspring. NOS: New Offspring Spouse. LE4: Life’s essential 4 score (four health behaviors, i.e., diet, physical activity, nicotine exposure, and sleep health). VCTE: vibration-controlled transient elastography. CVD: cardiovascular disease. BMI: body mass index. LE8: Life’s essential 8 score. Mild alcohol intake: ≤140 g/week for women or ≤210 g/week for men. Moderate alcohol intake: 141–350 g/week for women or 211–420 g/week for men.

We included participants from the FHS Generation 2 and Generation 3-based cohorts who underwent vibration-controlled transient elastography (VCTE) liver measurements [23]. Participants from Generation 2 (Framingham Offspring [FOS] and multi-ethnic OMNI-1) cohorts underwent VCTE at their tenth and fifth exam cycles, respectively, between 2019 and 2022. Generation 3-based participants including Third Generation, OMNI-2 and New Offspring Spouse (NOS) cohorts underwent VCTE during their third exam cycle between 2016 and 2019 [24]. From the combined Generation 2 and Generation 3 cohorts (n = 5219), we sequentially excluded participants with: missing LE4 health behavior components, incomplete or invalid VCTE data, missing education and alcohol intake data, a history of prevalent CVD (defined as coronary artery disease, heart failure, peripheral artery disease, and stroke) or non-skin cancer (other than non-melanoma skin cancer) and severe alcohol intake (>350 g/week for women and >420 g/week for men). The final group for analysis included 2962 participants (Figure 1). We further stratified the group by alcohol intake into mild (≤140 g/week for women; ≤210 g/week for men; n = 2704) and moderate (141–350 g/week for women; 211–420 g/week for men; n = 258) alcohol intake groups. From the final group (n = 2962), we also derived a group of participants that combined both mild and moderate alcohol intake with complete data on all LE8 components (n = 2841; referred to as “combined group”), to examine associations between LE8 and hepatic fibrosis (Figure 1). All participants provided informed consent, and the protocol for this study was approved by the Institutional Review Board (IRB) of the Boston University Medical Center (IRB H-32132,18 March 2016) and conducted in accordance with the U.S. Federal Policy for the Protection of Human Subjects (Common Rule).

### 2.2. Life’s Essential 8 (LE8) and Life’s Essential 4 (LE4) Scores

The LE8 score was defined according to the updated 2022 Presidential Advisory from the AHA [11] with minor modifications to adapt FHS data to the AHA LE8 criteria (Appendix Table A1) [25,26,27,28]. The LE8 comprises eight components: four health behaviors (collectively referred to as LE4 throughout this manuscript) and four health factors. Each component is scaled from 0 to 100, and the LE8 score is calculated as the unweighted average of all eight component scores. The total score ranges from 0 to 100, where higher values reflect better CVH. The LE4 score is the unweighted average of the four health behavior components. LE8 and LE4 scores were categorized as poor (0–49), intermediate (50–79), and ideal (≥80) CVH. Scores were also categorized as Low and High using the median as the cutoff. The LE4 score assesses diet quality (DASH-style dietary pattern), physical activity (minutes of moderate-to-vigorous activity per week), nicotine exposure (self-reported smoking status and cessation history), and sleep health (average hours of sleep per night). These components were assessed using validated questionnaires and self-reported measures. The health factors include BMI, non-HDL cholesterol, blood glucose (fasting glucose or glycated hemoglobin [HbA1c]), and blood pressure, which were assessed during in-person study visits using standardized measurements and laboratory testing. Additional details on the assessment of health behaviors and health factors are provided in Appendix A. Although the LE4 score was constructed to minimize direct overlap with the CMRFs used to define MASLD and MetALD, residual indirect overlap through shared metabolic pathways cannot be entirely excluded, and findings should be interpreted accordingly.

### 2.3. Hepatic Steatosis, Fibrosis, MASLD and MetALD

Hepatic steatosis and fibrosis were assessed by VCTE using a controlled attenuation parameter (CAP) and liver stiffness measurement (LSM), respectively. Measurements were performed by a certified operator using the FibroScan^®^ 502 Touch (Echosens, Paris, France), with M or XL probes selected based on the device’s probe selection tool, as previously described [29]. All participants fasted for at least four hours prior to the exam. A minimum of 10 valid measurements were obtained per participant, and the device automatically calculated the median CAP and LSM values along with the interquartile range (IQR). LSM results with an IQR-to-median ratio >0.30 were considered invalid [30]. Hepatic steatosis was defined as a CAP ≥274 dB/m and significant hepatic fibrosis as an LSM ≥8.2 kPa [31,32,33]. Throughout the manuscript, the term hepatic fibrosis refers specifically to significant hepatic fibrosis.

MASLD was defined as the presence of hepatic steatosis with mild alcohol intake (≤140 g/week for women or ≤210 g/week for men) and at least one of the following five CMRFs: (1) BMI ≥25 kg/m^2^ or waist circumference >80 cm in women or >94 cm in men; (2) blood pressure ≥130/85 mmHg or use of antihypertensive medication; (3) fasting glucose ≥100 mg/dL, HbA1c ≥5.7%, a diagnosis of T2DM, or treatment for T2DM; (4) triglycerides ≥150 mg/dL or use of lipid-lowering therapy; or (5) HDL cholesterol ≤50 mg/dL in women or ≤40 mg/dL in men, or use of lipid-lowering therapy [3]. MetALD was defined as MASLD in the setting of moderate alcohol intake (141–350 g/week for women or 211–420 g/week for men) [3]. Definitions of CMRFs are shown in Appendix Table A2.

### 2.4. Genetic Risk Score (GRS)

A weighted GRS was calculated based on 17 single nucleotide polymorphisms previously associated with hepatic steatosis and fibrosis [19,20] (Appendix Table A3). Genotyping in the FHS was performed with the Affymetrix 550 K Array and imputed to the 1000 Genomes Project reference panel [34]. Single nucleotide polymorphisms with imputation quality R^2^ > 0.5 and minor allele frequency >0.005 were selected for the GRS calculation. Each variant was weighted according to its published effect size (GOLD-weighted approach), and the cumulative GRS was computed as the sum of these weighted alleles. This GRS was derived from external studies [19,20] and applied in our cohort to assess effect modification rather than for risk prediction or validation. To assess effect modification of the association between CVH and hepatic outcomes by the GRS, participants were dichotomized into two groups based on the median GRS, representing low and high genetic risk for hepatic steatosis and fibrosis.

### 2.5. Covariates

We included age, sex, education level (no high school, high school, some college, and college degree), cohort (to adjust for familial relatedness), and alcohol intake as covariates in all models.

### 2.6. Statistical Analysis

We used multivariable-adjusted logistic regression models to relate the LE8 score with hepatic fibrosis in the combined group, and the LE4 score with MASLD and MetALD in the mild and moderate alcohol use groups, respectively (separate models for MASLD and MetALD). The LE4 and LE8 scores were used as continuous variables (per 10-point increment) and as categorical variables, comparing the intermediate and ideal categories to the poor category (reference group). Hepatic fibrosis, MASLD, and MetALD were modeled as binary outcomes (presence vs. absence, separate model for each).

All analyses were conducted using SAS software (version 9.4; SAS Institute Inc., Cary, NC, USA), with a two-sided *p*-value < 0.05 considered statistically significant. The LE4-MASLD and LE4-MetALD models were adjusted for age, sex, education level, and cohort. The LE8-hepatic fibrosis models were further adjusted for alcohol intake. We also examined whether the associations of LE4 with MASLD and MetALD, and of LE8 with fibrosis, were modified by genetic risk as captured by a GRS.

### 2.7. Secondary Analyses

We additionally assessed the relation of the individual components comprising LE8 with the respective outcomes (separate models for each exposure and outcome). Lastly, we cross-classified LE4 with the GRS using the respective median cutoffs to create four groups as follows: Low LE4–High GRS (referent), Low LE4–Low GRS, High LE4–High GRS, and High LE4–Low GRS. We examined the relation between these 4 groups and MASLD and MetALD (separate model for each outcome). The same categorization approach was used to evaluate the association between LE8/GRS groups and hepatic fibrosis. We also tested the associations of continuous and categorical LE8 with hepatic fibrosis among participants with mild alcohol use.

## 3. Results

Characteristics of the study groups are shown in Table 1. From the initial 2962 participants for LE4 analysis, 2704 (91%) had mild alcohol intake and 258 (9%) had moderate alcohol intake. Among those with mild alcohol intake, the prevalence of MASLD was 34% (n = 922), and 9% (n = 240) had hepatic fibrosis. Among those with moderate alcohol intake, the prevalence of MetALD was 40% (n = 102), and 8% (n = 21) had hepatic fibrosis. Compared to participants with mild alcohol intake, those with moderate alcohol intake had lower median LE8 and LE4 scores, higher CAP values, and a greater prevalence of current smoking. There were 2841 participants in the combined group, of whom 9% (n = 243) had hepatic fibrosis. The median alcohol intake in the combined group was 3 drinks per week. The median LSM was 5.0 kPa across the mild alcohol intake, moderate alcohol intake, and combined groups. A total of 91% of participants in the mild alcohol group and 94% in the moderate alcohol group had at least one cardiometabolic risk factor.

### 3.1. Association of LE4 with MASLD and MetALD

**Figure 2:**
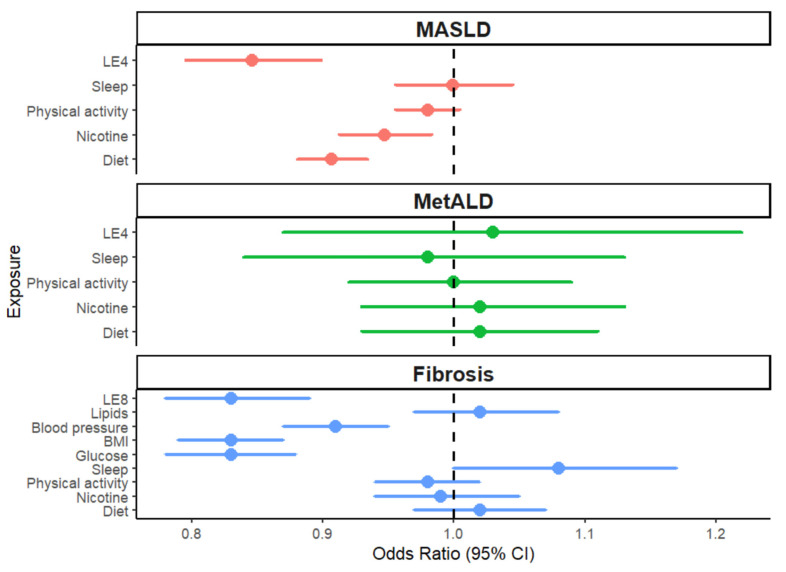
Associations of Cardiovascular Health with MASLD, MetALD, and Hepatic Fibrosis. The ORs of MASLD and MetALD are expressed per 10-point increase in LE4 and its components, and the ORs for hepatic fibrosis are expressed per 10-point increase in LE8 and its components. Fibrosis refers to significant hepatic fibrosis (Liver stiffness measurement ≥8.2 kPa). LE4, Life’s Essential 4; MASLD, Metabolic dysfunction-associated steatotic liver disease; MetALD, Metabolic dysfunction and alcohol-associated liver disease; OR, odds ratio; CI, confidence interval.

Table 2 and Figure 2 display the association of LE4 and its components with MASLD and MetALD. In multivariable models adjusted for age, sex, education, and cohort, each 10-point increase in LE4 was associated with 16% lower odds of MASLD. Participants with ideal LE4 had 54% lower odds of MASLD compared to those with poor LE4, while those with intermediate LE4 had 34% lower odds of MASLD. Among the individual LE4 components, higher scores in diet and nicotine exposure (i.e., healthier diet and less exposure to nicotine) were both related to lower odds of MASLD. We did not observe significant associations of sleep health or physical activity with MASLD.

We did not observe significant associations of the LE4 score or its individual components with MetALD (Table 2).

### 3.2. Association of LE8 with Hepatic Fibrosis

In models adjusted for age, sex, education, alcohol intake, and cohort, higher LE8 scores were significantly associated with lower odds of hepatic fibrosis (Figure 2, Table 3). Each 10-point increase in LE8 score was associated with 17% lower odds of hepatic fibrosis. Participants with intermediate and ideal LE8 scores had 37% and 42% lower odds of hepatic fibrosis, respectively, compared to those with poor LE8 scores. The association of LE8 score with lower odds of hepatic fibrosis persisted when the group was restricted to those with mild alcohol use.

Among individual LE8 components, higher scores in BMI, blood glucose, and blood pressure components (i.e., healthier BMI and lower values of blood glucose and blood pressure) were strongly associated with lower odds of hepatic fibrosis. In contrast, we did not observe significant associations of diet, nicotine exposure, physical activity, or non-HDL cholesterol components with odds of hepatic fibrosis. Interestingly, we observed a modest direct association between sleep health score and odds of hepatic fibrosis.

### 3.3. Effect Modification of the Genetic Risk Score on the Association of CVH with MASLD, MetALD, and Hepatic Fibrosis

We used the median values for LE4, LE8, and the GRS to create groups based on low/high values of each variable, and the distribution of participants in these groups is shown in Table 1. Among participants with mild alcohol use, we did not observe a difference in odds of MASLD between those in the Low LE4-Low GRS group and those in the Low LE4–High GRS group (referent) (Table 4). In contrast, both high LE4 categories (regardless of their GRS status) were significantly associated with lower odds of MASLD compared to the reference group.

In the moderate alcohol group, participants in the Low LE4–Low GRS and High LE4-Low GRS groups had significantly lower odds of MetALD compared to the reference group (Table 4). We did not observe differences in odds of hepatic fibrosis between any of the groups compared to the referent.

## 4. Discussion

In a community-based group of middle-aged and older adults, we observed that better cardiovascular health (CVH), as captured by Life’s Essential 4 (LE4) and Life’s Essential 8 (LE8) scores, was associated with lower liver disease burden. Higher LE4 scores were associated with substantially lower odds of MASLD but not MetALD, with diet and nicotine exposure showing the strongest associations. Higher LE8 scores were associated with lower odds of hepatic fibrosis, driven primarily by BMI, glucose, and blood pressure, whereas most behavioral components were not individually associated with hepatic fibrosis. We did not observe any effect modification by genetic risk on these associations.

Our findings support the notion that CVH and liver health are closely intertwined, and LE8 and its components can provide meaningful information about odds of steatosis and hepatic fibrosis beyond traditional liver-specific measures. The strong inverse association between LE4 and MASLD indicates that even a concise, behavior-focused metric can effectively distinguish individuals at higher versus lower odds of MASLD, whereas the absence of association between LE4 and MetALD in our study may reflect the dominant influence of alcohol exposure in this group, the smaller sample size, or the possibility that lifestyle-related factors play a comparatively lesser role among people with higher alcohol intake. These results are consistent with prior studies showing that better cardiovascular health is associated with lower risk of steatotic liver disease and hepatic fibrosis, while also addressing key limitations of earlier work [13,14,15,16,17,35,36]. Unlike previous analyses that relied on modified or incomplete CVH metrics, surrogate indices of steatosis such as fatty liver index [15,16], or outdated definitions [14], our study employed the full LE8 construct, and direct VCTE-based assessment of hepatic steatosis and fibrosis, providing measures that are more aligned with current clinical practice. By further stratifying participants by alcohol intake, we distinguished MASLD from MetALD, and characterized their distinct behavioral and metabolic profiles, providing a more precise and comprehensive understanding of how CVH may influence hepatic outcomes.

The finding that diet and nicotine exposure were the dominant LE4 components associated with MASLD is consistent with established links between dietary quality [37,38,39], smoking [40,41], and metabolic liver disease. The observed lower odds of MASLD with the individual diet component are consistent with prior studies showing that higher DASH diet scores are associated with a lower prevalence of MASLD [42,43]. Similarly, the strong associations of BMI, blood glucose, and blood pressure with hepatic fibrosis, a more advanced and clinically consequential stage of liver disease, support the concept that hepatic fibrosis reflects the cumulative effects of chronic metabolic stress. Visceral obesity drives hypertension and promotes insulin resistance, the latter being a key driver of systemic inflammation, type 2 diabetes, and hepatic steatosis [44,45]. In addition, the coexistence of type 2 diabetes and MASLD synergistically heightens cardiovascular disease risk and mortality [46,47,48]. Clinically, this may suggest that optimizing core cardiometabolic risk factors may be associated with cardiovascular and liver health, reinforcing the need for integrated care models that address both systems rather than treating them in isolation.

Prior studies have reported lower odds of hepatic fibrosis with higher diet quality. In one study including FHS and NHANES participants, however, this association was substantially attenuated after adjustment for hepatic fat content and BMI [49]. Another study in an NHANES population also observed lower odds of fibrosis with healthier dietary patterns, though fibrosis was assessed using the NAFLD fibrosis score rather than VCTE [43]. While we did not observe an individual association with the diet component, this difference may be partly explained by these methodological differences. The modest direct association between sleep health scores and odds of hepatic fibrosis is intriguing and counterintuitive, which could reflect residual confounding or misclassification in the sleep health metric. In addition, the LE8 sleep health component captures sleep duration but not sleep quality or disorders (e.g., sleep apnea), which may inadequately represent sleep-related odds of hepatic fibrosis in this population and warrants further exploration [50,51]. This finding should be interpreted cautiously and would benefit from replication before drawing pathophysiologic inferences.

The cross-classification of LE4 and LE8 with GRS provides further insight into how health behaviors and inherited genetic risk interact in terms of their relation to liver outcomes. Among participants with mild alcohol use, high LE4 scores were associated with lower odds of MASLD irrespective of genetic risk, whereas simply having a low-risk GRS in the context of poor LE4 did not meaningfully reduce odds of MASLD. This pattern suggests that favorable lifestyle and behavioral profiles may be associated with lower phenotypic expression of genetic risk, whereas low genetic risk does not fully compensate for adverse lifestyle. Our findings align with prior studies that show that while a high-risk GRS increases risk of steatosis, the effect is modest relative to the influence of poor lifestyle factors [18,19,20,21,22]. The GRS captures inherited variation in pathways central to hepatic lipid metabolism, fibrogenesis, and insulin signaling, including well-established variants such as PNPLA3, TM6SF2, and MBOAT7, that collectively influence individual susceptibility to steatosis and fibrosis. These observations are similar to studies on cardiovascular genetics, where high polygenic risk can be partly offset by favorable lifestyle, and individuals with high polygenic risk derive disproportionate benefit from intensive risk factor modification [52,53].

Our findings, in the context of the broader literature, inform several potential applications. First, both CVH scores (LE4 and LE8) could help identify individuals with “low” alcohol intake who nonetheless are at higher odds of liver disease due to poor cardiometabolic health. Second, CVH metrics may help prioritize patients for non-invasive liver assessment (e.g., VCTE) in primary care settings. Third, our findings build on observations that better CVH reduces the odds of MASLD, even in individuals with high-risk genetic variants. However, the clinical utility of GRS remains uncertain, and it should currently be interpreted as a marker of underlying susceptibility rather than a clinical decision-making tool, underscoring the need for further validation in diverse populations.

## 5. Strengths and Limitations

Key strengths of this investigation include a well-characterized, community-based group with detailed phenotyping of CVH, alcohol use, and liver outcomes; the parallel assessment of MASLD/MetALD and hepatic fibrosis; and integration of genetic risk with standardized CVH metrics. Diagnoses of MASLD and MetALD were based on CAP and LSM measurements obtained via VCTE, a widely used clinical tool endorsed by current guidelines.

Limitations include the cross-sectional design, which precludes causal inference and temporal assessment; limited generalizability given the cohort’s middle-aged to older age range and predominantly European ancestry profile, which restricts extrapolation to younger and more ethnically diverse populations, and reliance on self-reported alcohol, diet, physical activity, and sleep health, which may introduce misclassification and recall bias. Despite the use of standardized, previously validated protocols for data collection, widely accepted in epidemiologic research (Appendix A), inherent limitations remain, including imprecision in dietary assessment, potential overestimation of physical activity, and incomplete capture of sleep quality. While we could not directly quantify the magnitude of misclassification from self-reported LE8 components, any such misclassification is likely non-differential with respect to liver outcomes and would bias estimates toward the null. Accordingly, the observed associations between LE8/LE4 scores and liver outcomes are likely conservative. Education level was included as a proxy for socioeconomic status; however, more granular measures such as income and neighborhood deprivation were unavailable. Comorbidities independently associated with liver disease (e.g., hypothyroidism, obstructive sleep apnea) and use of metabolically active medications (e.g., statins, metformin, glucocorticoids) beyond those captured within CMRF definitions were not fully accounted for, representing additional sources of residual confounding that could bias estimates in either direction. Residual confounding from other unmeasured environmental factors remains possible. Case counts for MetALD and hepatic fibrosis, particularly within genetic strata, were modest, reducing precision and power. Although liver biopsy remains the histologic gold standard, its invasive nature and sampling variability render it impractical in large epidemiologic cohorts; VCTE-based CAP and LSM measurements are widely validated, guideline-endorsed, and have demonstrated prognostic accuracy comparable to biopsy for predicting liver-related clinical outcomes [54].

## 6. Conclusions

Better CVH was associated with lower odds of MASLD and hepatic fibrosis. Future studies are warranted to assess how longitudinal changes in CVH metrics relate to incident liver disease including MetALD.

## Figures and Tables

**Table 1:** Baseline Characteristics of the study groups. Values are represented as mean (standard deviation [SD]), unless otherwise specified.

Characteristic	Mild Alcohol Use (n = 2704)	Moderate Alcohol Use (n = 258)	Combined ^a^ (n = 2841)
Women, n (%)	1558 (58)	128 (50)	1635 (58)
Age, years	58 (12)	58 (11)	58 (12)
Waist, inches	39 (6)	39 (5)	39 (6)
BMI, kg/m^2^	28 (5)	27 (5)	28 (5)
Fasting blood glucose, mg/dL; median (Q1, Q3)	97 (90, 104)	98 (92, 105)	97 (90, 103)
T2DM n (%)	229 (8)	20 (8)	140 (5)
Treatment for diabetes, n (%)	166 (6)	9 (3)	111 (4)
SBP, mmHg	121 (15)	123 (14)	121 (15)
DBP, mmHg	74 (9)	76 (9)	75 (9)
Treatment for hypertension, n (%)	739 (27)	87 (34)	746 (26)
Triglycerides, mg/dL; median (Q1, Q3)	91 (67, 132)	86 (68, 130)	90 (67, 130)
HDL cholesterol, mg/dL; median (Q1, Q3)	57 (46, 71)	69 (54, 83)	58 (47, 72)
Total cholesterol, mg/dL; median (Q1, Q3)	184 (162, 209)	200 (176, 220)	187 (165, 211)
Lipid-lowering medication, n (%)	771 (29)	71 (28)	760 (27)
Smoking status, n (%)	123 (5)	29 (11)	140 (5)
Alcohol intake, drinks/week	2 (0, 6)	17 (14, 21)	3 (0, 7)
Alcohol intake category, n (%)			
Mild ^b^	2704 (100)	0	2600 (92)
Moderate ^c^	0	258 (100)	241 (8)
Heavy drinking history, yes, n (%)	44 (2)	39 (15)	77 (3)
Weighted alcohol intake history, drinks/year; median (Q1, Q3)	48 (12, 100)	216 (157, 338)	55 (15, 123)
Education level, n (%)			
No high school	21 (1)	1 (<1)	20 (1)
High school	335 (12)	36 (14)	347 (12)
Some college	738 (27)	77 (30)	773 (27)
College degree	1610 (60)	144 (56)	1701 (60)
**Cardiovascular Health**			
LE4 Score; median (Q1, Q3)	67.5 (57.5, 77.5)	66.3 (57.8, 76.3)	67.5 (57.5, 77.5)
LE4 Score Category, n (%)			
Poor	301 (11)	45 (17)	320 (11)
Intermediate	1814 (67)	170 (66)	1904 (67)
Ideal	589 (22)	43 (17)	617 (22)
LE8 Score; median (Q1, Q3)	75.0 (64.4, 88.8)	71.9 (61.9, 84.4)	74.4 (64.4, 88.8)
LE8 Score category, n (%)			
Poor	201 (7)	34 (13)	114 (4)
Intermediate	1457 (54)	151 (59)	1608 (57)
Ideal	1046 (39)	73 (28)	1119 (39)
**VCTE Measurements**			
LSM, kPa; median (Q1, Q3)	5.0 (4.2, 6.2)	5.1 (4.1, 6.2)	5.0 (4.2, 6.2)
CAP, dB/m	256.5 (53.9)	264.5 (52.1)	255.8 (53.1)
Steatosis (CAP ≥274 dB/m), n (%)	927 (34)	104 (40)	959 (34)
Hepatic fibrosis (LSM ≥8.2 kPa), n (%)	240 (9)	21 (8)	243 (9)
MASLD, n (%)	922 (34)	0	861 (30)
MetALD, n (%)	0	102 (40)	91 (3)
**Cardiometabolic Risk Factors, n (%)**			
Abdominal obesity	2311 (85)	229 (89)	2427 (85)
Impaired glucose metabolism	1170 (43)	120 (47)	1176 (41)
High blood pressure	870 (32)	104 (40)	890 (31)
Hypertriglyceridemia	1050 (39)	99 (38)	1055 (37)
Low HDL	1124 (42)	82 (32)	1107 (39)
**Number of Cardiometabolic Risk Factors, n (%)**			
0	247 (9)	15 (6)	260 (9)
1	725 (27)	65 (25)	788 (28)
2	524 (20)	68 (26)	583 (21)
3	435 (16)	42 (16)	463 (16)
4	418 (15)	33 (13)	423 (15)
5	355 (13)	35 (14)	324 (11)
**CVH Score and GRS Categories, n (%) ^d^**			
Low LE4–High GRS (Referent Group)	666 (25)	65 (24)	
Low LE4–Low GRS	854 (32)	69 (27)	
High LE4–High GRS	497 (18)	53 (21)	
High LE4–Low GRS	687 (25)	71 (28)	
			
Low LE8–High GRS (Referent Group)			910 (32)
Low LE8–Low GRS			1138 (40)
High LE8–High GRS			319 (11)
High LE8–Low GRS			474 (17)

**Abbreviations**: BMI, body mass index; T2DM, Type 2 diabetes; SBP, systolic blood pressure; DBP, diastolic blood pressure; HDL, high-density lipoprotein; LE4, Life’s Essential 4; LE8, Life’s Essential 8; VCTE, vibration-controlled transient elastography; LSM, liver stiffness measurement; kPa, kilopascal; CAP, controlled attenuation parameter; MASLD, metabolic dysfunction-associated steatotic liver disease; MetALD, metabolic and alcohol-related liver disease; GRS, genetic risk score. ^a^ The combined group for hepatic fibrosis analyses included individuals with mild and moderate alcohol use after excluding 121 participants due to missing LE8 components data. ^b^ ≤140 g/week for women and ≤210 g/week for men. ^c^ 141–350 g/week for women and 211–420 g/week for men. ^d^ The cutoffs of the low and high categories were the median values of the LE4 or LE8 scores and GRS.

**Table 2:** Associations of continuous and categorical LE4 with MASLD and MetALD.

	MASLD (n = 922)			MetALD (n = 102)		
	OR	95% CI	*p*-Value	OR	95% CI	*p*-Value
**Continuous LE4 model, per 10-unit increase**	0.84	(0.80–0.90)	<0.001	1.03	(0.87–1.22)	0.76
**LE4 Components, per 10-unit increase**						
Diet	0.91	(0.88–0.93)	<0.001	1.02	(0.93–1.11)	0.71
Nicotine exposure	0.95	(0.91–0.98)	0.004	1.02	(0.93–1.13)	0.65
Physical activity	0.98	(0.96–1.01)	0.12	1.00	(0.92–1.09)	0.93
Sleep Health	0.99	(0.96–1.05)	0.97	0.98	(0.84–1.13)	0.73
**Categorical LE4 model** ^a^						
Intermediate vs. Poor	0.66	(0.51–0.87)	0.002	1.09	(0.51–2.34)	0.82
Ideal vs. Poor	0.46	(0.33–0.63)	<0.001	1.09	(0.41–2.87)	0.87

Abbreviations: LE4, Life’s Essential 4; MASLD, Metabolic dysfunction-associated steatotic liver disease; MetALD, Metabolic dysfunction and alcohol-associated liver disease; OR, odds ratio; CI, confidence interval. All models were adjusted for age, sex, education, and cohort. ^a^ LE4 scores were categorized into poor (0–49), intermediate (50–79), and ideal (≥80).

**Table 3:** Association of continuous and categorical LE8 with hepatic fibrosis.

	Hepatic Fibrosis ^a^ (n = 243)		
	OR	95% CI	*p*-Value
**Continuous LE8 model, per 10-unit increase**	0.83	(0.78–0.89)	<0.001
**LE8 components, per 10-unit increase**			
Blood glucose	0.83	(0.78–0.88)	<0.001
BMI	0.83	(0.79–0.87)	<0.001
Blood pressure	0.91	(0.87–0.95)	<0.001
Physical activity	0.98	(0.94–1.02)	0.24
Nicotine exposure	0.99	(0.94–1.05)	0.78
Non-HDL Cholesterol	1.02	(0.97–1.08)	0.42
Diet	1.02	(0.97–1.07)	0.46
Sleep Health	1.08	(1.00–1.17)	0.04
**Categorical LE8 ^b^**			
Intermediate vs. Poor	0.63	(0.46–0.86)	0.003
Ideal vs. Poor	0.58	(0.41–0.82)	0.002

Abbreviation: LE8 Life’s Essential 8; OR, odds ratio; CI, confidence interval; LSM, liver stiffness measurement; BMI, body mass index; DASH, Dietary Approaches to Stop Hypertension diet. All models were adjusted for age, sex, education, alcohol intake, and cohort. ^a^ Hepatic fibrosis was defined as an LSM ≥8.2 kPa. ^b^ LE8 scores were categorized into poor (0–49), intermediate (50–79), and ideal (≥80).

**Table 4:** Effect of Genetic Risk Score on Associations of Cardiovascular Health with MASLD, MetALD, and Hepatic Fibrosis.

		MASLD (n = 922) ^a^			MetALD (n = 102) ^a^			Hepatic Fibrosis ^b^ (n = 243) ^a^		
Categories		OR	95% CI	*p*-Value	OR	95% CI	*p*-Value	OR	95% CI	*p*-Value
CVH ^c^	GRS									
Low	High	Reference			Reference			Reference		
Low	Low	0.91	(0.73, 1.13)	0.40	0.41	(0.20, 0.87)	0.02	0.90	(0.65, 1.23)	0.50
High	High	0.70	(0.54, 0.90)	0.01	0.73	(0.34, 1.60)	0.43	1.19	(0.76, 1.85)	0.44
High	Low	0.57	(0.45, 0.73)	<0.001	0.42	(0.20, 0.86)	0.02	0.85	(0.55, 1.29)	0.44

**Abbreviations**: CVH, Cardiovascular Health; LE4, Life’s Essential 4; GRS, Genetic Risk Score; MASLD, Metabolic dysfunction-associated steatotic liver disease; MetALD, Metabolic and alcohol-associated liver disease; OR, odds ratio; CI, confidence interval. ^a^ Number of participants with complete GRS and LE4 or LE8 data for analysis in each group. ^b^ Hepatic fibrosis was defined as significant fibrosis with an LSM ≥8.2 kPa. ^c^ CVH was assessed using LE4 for the MASLD and MetALD groups, and LE8 for the hepatic fibrosis group.

## Data Availability

The original contributions presented in this study are included in the article. Further inquiries can be directed to the corresponding author.

## Abbreviations

The following abbreviations are used in this manuscript:

MASLD	Metabolic dysfunction-associated steatotic liver disease
NAFLD	Non-alcoholic fatty liver disease
CMRFs	Cardiometabolic risk factors
BMI	Body mass index
WC	Waist circumference
BP	Blood pressure
TG	Triglycerides
HDL	High-density lipoprotein
MetALD	Metabolic and alcohol-associated liver disease
CVH	Cardiovascular health
AHA	American Heart Association
LS7	Life’s Simple 7
LE8	Life’s Essential 8
CVD	Cardiovascular disease
LE4	Life’s Essential 4
FHS	Framingham Heart Study
GRS	Genetic risk score
VCTE	Vibration-controlled transient elastography
FOS	Framingham Offspring Study
OMNI	Multi-ethnic OMNI cohort
NOS	New Offspring Spouse cohort
CAP	Controlled attenuation parameter
LSM	Liver stiffness measurement
IQR	Interquartile range
T2DM	Type 2 diabetes mellitus
HbA1c	Glycated hemoglobin

## Appendix A

### Appendix A.1. Details on Assessment of Life’s Essential 8 (LE8) Components

Height and weight were measured using standardized protocols at each respective exam. BMI was calculated as measured weight in kilograms divided by the square of height in meters (kg/m^2^). Waist circumference (in centimeters) was measured at the level of the umbilicus. The average of two measurements (5 min apart) using a mercury column sphygmomanometer was used to capture systolic and diastolic blood pressure. Routine phlebotomy protocols were used for measurement of blood lipids, blood glucose, and hemoglobin A1c (HbA1c). Non-HDL-C was defined as subtracting HDL-C from total cholesterol. Information on the use of lipid lowering, antidiabetic, and antihypertensive medications in the last year was collected at each exam.

### Appendix A.2. Physical Activity

Physical activity was assessed through number of minutes spent engaging in moderate or intense activity. This value was then scored according to AHA guidelines.

### Appendix A.3. Nicotine Exposure

Nicotine exposure was captured through self-reported smoking status, including current or former use and years since quitting. Data on secondhand smoke were not available.

### Appendix A.4. Diet

The diet assessment was performed using the validated semi-quantitative 126-item Harvard food frequency questionnaire (FFQ). Diet quality was assessed using a DASH-style scoring system based on quantiles of DASH-style diet adherence. For the Framingham Offspring cohort, DASH components from Exam 9 were used as a proxy for Exam 10, and for the Omni 1 cohort, DASH components from Exam 4 were used as a proxy for Exam 5.

### Appendix A.5. Sleep Health

Sleep health was defined as self-reported average hours of sleep per night.

**Table A1:** Life’s Essential 8 Score Definition and FHS data adaptation.

Domain	Metric	Method of Measurement	Quantification of Metric as per AHA		Data Discrepancy in FHS and Solution
**Health** **Behaviors**	**Diet**	Quantiles of DASH-style diet adherence or HEI-2015 (population)	**Points**	**Level**	Adhered to AHA guidance; no data discrepancy
			100 points	≥95th percentile	
			80 points	75th–94th percentile	
			50 points	50th–74th percentile	
			25 points	25th–49th percentile	
			0 points	1st–24th percentile	
	**Physical** **Activity**	Minutes of moderate or vigorous physical activity per week	**Points**	**Minutes/week**	Most participants reported ≥150 min of moderate or intense physical activity per week and would therefore be scored 100 points. A physical activity index (PAI) was calculated using a weighted average of hours spent engaging in moderate or intense physical activity. The PAI was then categorized into seven quantile groups and mapped to the LE8 point system.
			100 points	≥150	
			90 points	120–149	
			80 points	90–119	
			60 points	60–89	
			40 points	30–59	
			20 points	1–29	
			0 points	0	
	**Nicotine** **Exposure**	Smoking status	**Points**	**Status**	No available data on secondhand smoke, therefore we did not subtract 20 points from any participant
			100 points	Never smoker	
			75 points	Former smoker, quit ≥5 yr	
			50 points	Former smoker, quit 1–<5 yr	
			25 points	Former smoker, quit <1 yr	
			0 points	Current smoker	
			Subtract 20 points for secondhand smoke		
	**Sleep Health**	Average hours of sleep per night	**Points**	**Hours**	Adhered to AHA guidance; no data discrepancy
			100 points	7–8	
			90 points	9	
			70 points	6	
			40 points	5 or ≥10	
			20 points	4	
			0 points	<4	
**Health** **Factors**	**BMI**	Body mass index	**Points**	**BMI (kg/m^2^)**	Adhered to AHA guidance; no data discrepancy
			100 points	<25	
			70 points	25–29.9	
			30 points	30–34.9	
			15 points	35–39.9	
			0 points	≥40	
	**Blood** **Glucose**	Fasting blood glucose or HbA1c	**Points**	**Level**	Adhered to AHA guidance; no data discrepancy
			100 points	No history of diabetes and FBG <100 mg/dL (or HbA1c <5.7%)	
			60 points	No diabetes and FBG	
			40 points	Diabetes with HbA1c <7.0%	
			30 points	Diabetes with HbA1c 7.0–7.9%	
			20 points	Diabetes with HbA1c 8.0–8.9%	
			10 points	Diabetes with HbA1c 9.0–9.9%	
			0 points	Diabetes with HbA1c ≥10.0%	
	**Blood** **Pressure**	Systolic and diastolic blood pressure	**Points**	**Level**	Adhered to AHA guidance; no data discrepancy
			100 points	<120/<80 mm/Hg	
			75 points	120–129/<80 mm/Hg	
			50 points	130–139 or 80–89 mm/Hg	
			25 points	140–159 or 90–99 mm/Hg	
			0 points	≥160 or ≥100 mm/Hg	
			If drug treated level, subtract 20 points		
	**Blood** **Lipids**	Non-HDL cholesterol	**Points**	**Level**	Adhered to AHA guidance; no data discrepancy
			100 points	<130 mg/dL	
			60 points	130–159 mg/dL	
			40 points	160–189 mg/dL	
			20 points	190–219 mg/dL	
			0 points	≥220 mg/dL	
			If drug treated level, subtract 20 points		

Abbreviations: DASH, Dietary Approaches to Stop Hypertension; HEI, Healthy Eating Index; AHA, American Heart Association; PAI, physical activity index; LE8, Life’s Essential 8; BMI, body mass index; HbA1c, hemoglobin A1c; FBG, fasting blood glucose; mmHg, millimeters of mercury; HDL, high-density lipoprotein; non-HDL, non–high-density lipoprotein cholesterol; FHS, Framingham Heart Study.

**Table A2:** MASLD and MetALD Cardiometabolic Risk Factor Criteria and FHS Data Adaptation.

Cardiometabolic Risk Factor	Definition	Discrepancy in FHS	Solution or Adjustment Used
**Abdominal obesity**	BMI ≥25 kg/m^2^ (23 for Asians) or waist circumference >94 cm for males and >80 cm for females or ethnically adjusted	None	Adhered to the definition
**High blood pressure**	SBP ≥ 130 mm Hg and/or DBP ≥ 85 mm Hg or history of antihypertensive usage	None	Adhered to the definition
**Impaired glucose** **metabolism**	Fasting serum glucose ≥100 mg/dL or 2 h post-load glucose levels ≥140 mg/dL or HbA1c ≥5.7% or diagnosis of T2DM or treatment for T2DM	Data on 2 h post–load glucose levels or on the diagnosis history of T2DM were not available.	We used random blood glucose levels ≥200 mg/dL or fasting blood glucose ≥126 mg/dL, or HbA1c ≥5.7%, or treatment for T2DM to define the presence of T2DM.
**Hypertriglyceridemia**	Plasma triglycerides >150 mg/dL or use of lipid lowering treatment	N/A	Adhered to the definition
**Low HDL**	Plasma HDL ≤40 mg/dL for males and ≤50 mg/dL for females or use of lipid lowering treatment	N/A	Adhered to the definition

**Abbreviations**: MASLD, Metabolic dysfunction-associated steatotic liver disease; MetALD, metabolic and alcohol-associated liver disease; BMI, body mass index; SBP, systolic blood pressure; DBP, diastolic blood pressure; HbA1c, hemoglobin A1c; T2DM, type 2 diabetes mellitus; T2D, type 2 diabetes; HDL, high-density lipoprotein; FHS, Framingham Heart Study.

**Table A3:** Genes associated with hepatic steatosis and fibrosis included in the genetic risk score.

Genetic Included in the Genetic Risk Score			
Gene Symbol	Gene Description	Gene Symbol	Gene Description
*TOR1B*	Torsin family 1 member B	*PTPRD*	Receptor-type tyrosine-protein phosphatase delta
*FTO*	Fat mass and obesity associated	*GCKR*	Glucokinase regulator
*COBLL1/GRB14*	Cordon-bleu WH2 repeat protein like 1/Growth factor receptor-bound protein 14	*TRIB1*	Tribbles homolog 1
*INSR*	Insulin receptor	*GPAM*	Glycerol-3-phosphate acyltransferase
*SREBF1*	Sterol regulatory element-binding transcription factor 1	*MTARC1*	Mitochondrial amidoxime-reducing component 1
*PNPLA2*	Patatin-like phospholipase domain-containing protein 2	*MTTP*	Microsomal triglyceride transfer protein large subunit
*PNPLA3*	Patatin-like phospholipase domain-containing protein 3	*ADH1B*	Alcohol dehydrogenase 1B
*TM6SF2*	Transmembrane 6 superfamily 2	*TMC4/MBOAT7*	Transmembrane channel like 4/Membrane-bound O-acyltransferase domain containing 7
*APOE*	Apolipoprotein E		
